# Early Signaling in Primary T Cells Activated by Antigen Presenting Cells Is Associated with a Deep and Transient Lamellal Actin Network

**DOI:** 10.1371/journal.pone.0133299

**Published:** 2015-08-03

**Authors:** Kole T. Roybal, Emily M. Mace, Judith M. Mantell, Paul Verkade, Jordan S. Orange, Christoph Wülfing

**Affiliations:** 1 Department of Immunology, UT Southwestern Medical Center, Dallas, Texas, United States of America; 2 Children's Hospital of Philadelphia Abramson Research Center, University of Pennsylvania, Philadelphia, Pennsylvania, United States of America; 3 School of Biochemistry, University of Bristol, Bristol, United Kingdom; 4 Department of Cell Biology, UT Southwestern Medical Center, Dallas, Texas, United States of America; 5 School of Cellular and Molecular Medicine, University of Bristol, Bristol, United Kingdom; University of Iowa, UNITED STATES

## Abstract

Cellular signaling transduction critically depends on molecular interactions that are in turn governed by dynamic subcellular distributions of the signaling system components. Comprehensive insight into signal transduction requires an understanding of such distributions and cellular structures driving them. To investigate the activation of primary murine T cells by antigen presenting cells (APC) we have imaged more than 60 signaling intermediates during T cell stimulation with microscopy across resolution limits. A substantial number of signaling intermediates associated with a transient, wide, and actin-associated lamellum extending from an interdigitated T cell:APC interface several micrometers into the T cell, as characterized in detail here. By mapping the more than 60 signaling intermediates onto the spatiotemporal features of cell biological structures, the lamellum and other ones previously described, we also define distinct spatial and temporal characteristics of T cell signal initiation, amplification, and core signaling in the activation of primary T cells by APCs. These characteristics differ substantially from ones seen when T cells are activated using common reductionist approaches.

## Introduction

T cell activation occurs in cellular interactions between T cells and antigen-presenting cells (APC). During T cell activation signaling intermediates enrich in distinct locations at specific times within the cell [1–5]. Yet, studies with large numbers of signaling intermediates are missing and it is largely unresolved how such dynamic organization is related to underlying cytoskeletal structures. However, processes that regulate the cell-wide spatiotemporal organization of an entire signaling system have remained largely elusive. Here we characterize one such process.

The spatiotemporal organization of T cell activation on APCs is dynamic and complex [3]. Accumulation of molecules at the interface center (TCR, PKCθ) and in the periphery (LFA-1, actin) is long established [1, 2, 6]. In addition, in T cell activation by planar APC substitutes, the TCR and associated proximal signaling molecules coalesce into microclusters [7–9]. However, as cellular organization has been difficult to study at the system-scale in primary T cells activated by APC, it is still largely unclear how signaling is comprehensively organized in T cell/APC conjugates and which cellular structures drive the organization.

A powerful way to discover organizing principles in signal transduction is to analyze the spatiotemporal organization of the signaling network at the system-scale. Such analyses can elucidate higher-order mechanisms in the formation and resolution of signalling assemblies that are inaccessible to single gene/protein studies. To identify cellular processes controlling signaling organization, we have extended our live primary T cell:APC conjugate imaging data to more than 60 molecules involved in T cell activation and have furthered our understanding of the T cell signaling organization with microscopy across resolution limits. This system-scale imaging analysis of T cell signaling in response to APC stimulation revealed an actin-associated lamellum that organizes a substantial part of the T cell signaling system. By mapping a large and diverse set of signaling intermediates onto the spatiotemporal features of cell biological structures including the lamellum we define distinct spatial and temporal characteristics of T cell signal initiation, amplification, and core signaling in the activation of primary T cells by APCs.

## Results

### Interface accumulation of many signaling intermediates reaches deep into the T cell

**Fig 1:**
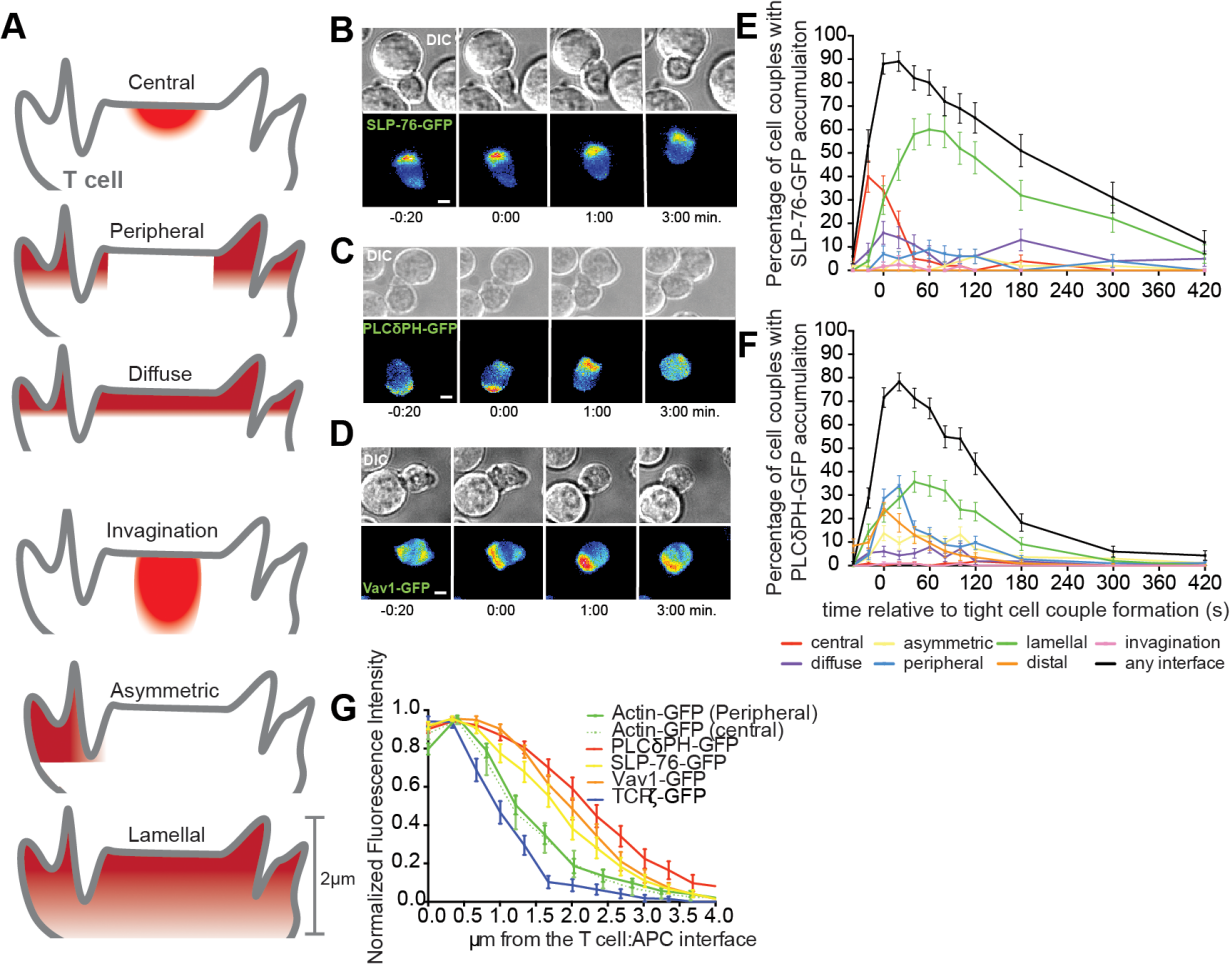
Critical regulators of T cell activation show transient localization to the T cell lamellum. **(A)** The panel represents six categories to classify spatiotemporal signaling features that reflect underlying cell biological structures. The APC above the T cell is not shown. Central reflects a large central signaling complex, peripheral the part of the actin network stabilizing the interface edge. Diffuse reflects cortical accumulation, invagination enrichment in a transient large T cell invagination suggested to contribute to early signal resetting [11]. Asymmetric reflects individual small lamella and the lamellal pattern is characterized here. (**B-D**) Representative interactions of 5C.C7 T cells expressing (B) SLP-76-GFP (S1 Video) (C) PLCδPH-GFP (PIP_2_ sensor)[3] or (D) Vav1-GFP (S2 Video) with peptide-loaded CH27s (10μM MCC) over the indicated time relative to formation of a tight cell conjugate are given. DIC images are shown on top and top-down maximum projections of 3D fluorescence data are shown in the bottom panels in a rainbow-like, false-color intensity scale (increasing from blue to red)(scale bar = 2μm). (**E, F**) 5C.C7 T cells expressing GFP-tagged signaling intermediates or sensors were stimulated with peptide loaded CH27 B cell lymphoma APCs (10μM MCC). The percentage of all T cells analyzed showing accumulation in defined patterns (A)[3] are shown for (E) SLP-76-GFP (number of cell couples analyzed across multiple independent experiments, n = 56) and (F) PLCδPH-GFP (PIP_2_ sensor) (n = 115). Vav1-GFP data are similarly quantified in Figure 3C of [10]. (**G**) Amounts relative to maximum as a function of the distance from the interface measured from live cell conjugates expressing GFP-actin (n = 15), PLCδPH-GFP (PIP_2_) (n = 18), SLP-76-GFP (n = 19), Vav1-GFP (n = 18) and TCRζ-GFP (n = 15) measured at the 1min time point across the entire interface are given. Error bars are s.e.m.

To gain insight into the organization of T cell signaling and the cellular structures driving it, we imaged T cell signaling via live cell fluorescence microscopy at a large scale. *In vitro* primed primary 5C.C7 TCR transgenic CD4^+^ T cells were retrovirally transduced to express fluorescently tagged signaling intermediates and sensors (> 60). Time-lapsed fluorescence microscopy was performed with transduced T cells activated by CH27 B cell lymphoma APCs pulsed with 10μM moth cytochrome C (MCC) antigenic peptide. This experimental setup provides an *in vitro* model for the reactivation of primed T cells, e.g. in the delivery of T cell help. 3D accumulation patterns at the T cell:APC interface (Fig 1A) were determined as previously established [3] and recently reviewed [10]. A region of signaling characterized by transient signaling intermediate accumulation originating across the entire T cell:APC interface and extending several micrometers into the T cell, the ‘lamellal pattern’ (Fig 1B–1D, S1 and S2 Videos), was prominent. The lamellal pattern was anecdotally observed in two earlier studies [3, 5] but remained largely uncharacterized. Molecules with dominant lamellal accumulation include the adaptor SH2 domain containing leukocyte phosphoprotein of 76 kDa (SLP-76), the phosphatidylinositol 4,5 bisphosphate (PIP_2_) sensor pleckstrin homology domain of phospholipase Cδ (PLCδPH), and the Rho family guanine nucleotide exchange factor Vav1 (Fig 1B–1F)(Figure 3C in [10]). Others are discussed below. Defining features of the lamellal pattern were its transience and the extension from the interface deep into the T cell. Prominent lamellal localization was largely restricted to the first three minutes of T cell coupling (Fig 1E and 1F)(Figure 3C in [10]), coinciding with the peak of biochemical signaling activity and translocation of transcription factors such as NFAT to the nucleus [3]. Measuring sensor fluorescence intensity as a function of the distance from the T cell:APC interface, lamellal signaling intermediates reached deeper into the T cell than the TCR as a mostly cell surface-localized molecule (Fig 1G).

### F-actin reaches deep into the T cell early during activation by APCs

**Fig 2:**
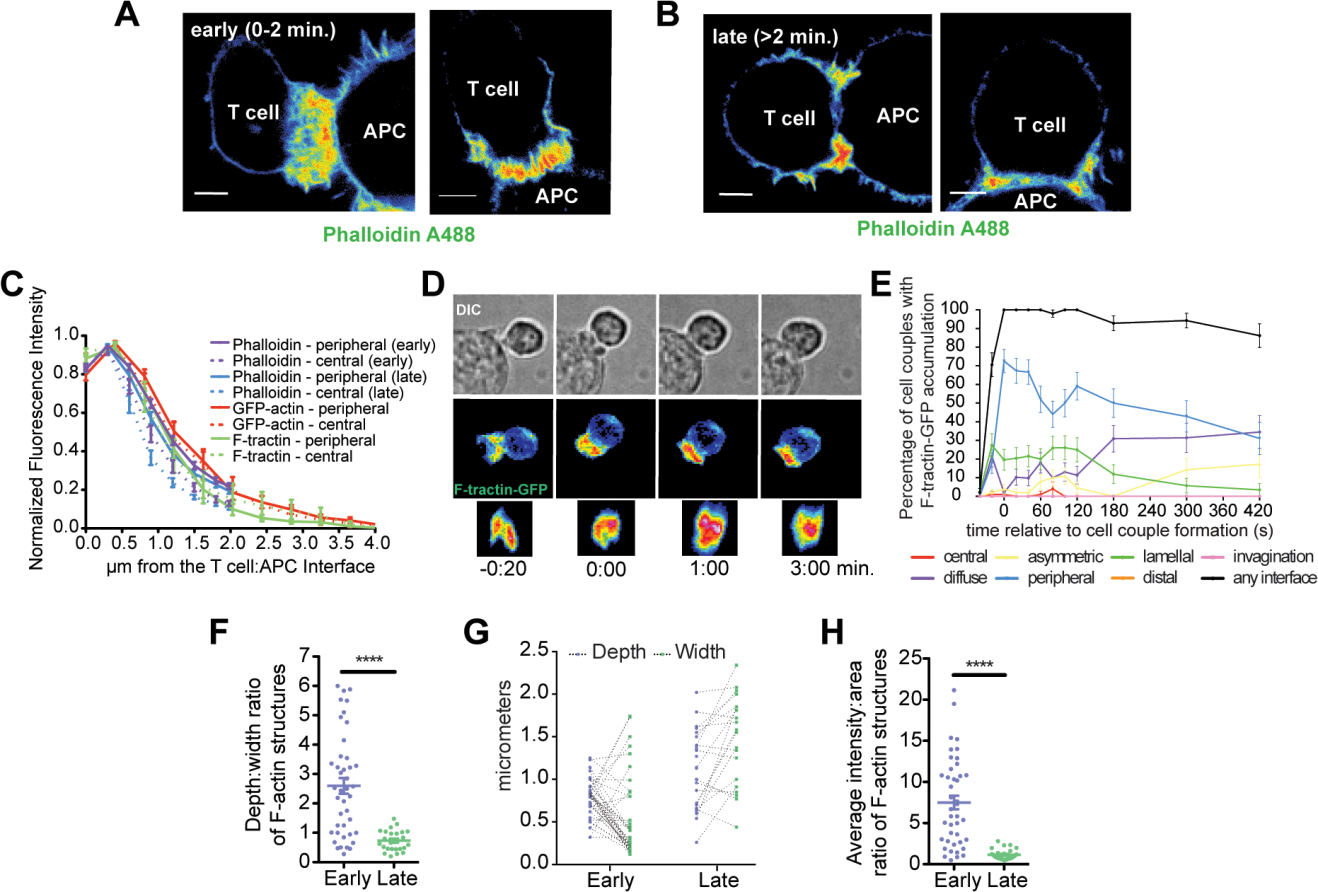
Actin displays transient lamellal accumulation. **(A, B)** Phalloidin stained 5C.C7 T cell:CH27 APC conjugates imaged by STED 1-2min (A) and >2min (B) after cell conjugate formation are shown in a rainbow-like color scale (scale bars = 2μm)(see methods for precise time point definition). (**C**) Actin amounts relative to maximum as a function of the distance from the interface in the periphery (outer 25% of the interface diameter) and center (middle 50% of the interface) measured from fixed Phalloidin stained 5C.C7 T cell:CH27 APC conjugates at an early (1-2min, number of cell couples analyzed across multiple independent experiments, n = 26) and late (2-5min, n = 16) time point (see methods for precise time point definition) by STED and live cell conjugates expressing GFP-actin (n = 15, same cells as in Fig 1G) or F-tractin-GFP (n = 19) (both measured at 1min) are given. (**D**) A representative interaction of a 5C.C7 T cell expressing F-tractin-GFP (S3 Video) is given similar to Fig 1B. In addition, an en face view of the T cell is shown at the bottom. (**E**) The pattern classification graph is given for F-tractin-GFP similar to Fig 1E (n = 51). **(F)** The depth to width ratio of resolved phalloidin stained F-actin structures (>135% above cellular background) in early (n = 12) and late (n = 12) STED imaged 5C.C7 T cell:CH27 APC conjugates is given (see S1 Fig for analysis details). **(G)** F-actin structure depth and width from F are given with dotted lines connecting paired measurements from the same structure for early and late cell conjugates. (**H**) The average intensity to area ratio of F-actin structures from F is given. Error bars are s.e.m. Significance was determined by Student’s t-test (*p<0.05, **p<0.001, ***p<0.0001).

As a wide actin sheet of comparable geometry to the lamellal pattern underlies the interface between natural killer cells and their targets [12, 13], we investigated T cell actin distributions. Various actin distributions have been previously described in T cell:APC couples, such as a peripheral actin ring, individual actin-rich protrusions, or cortical actin lining the plasma membrane [14]. Here we determined, whether a wide actin sheet matching the lamellal signaling distribution exists in addition to the established structures. Using stimulated emission depletion (STED) microscopy, 5C.C7 T cell conjugates with antigen pulsed (10μM MCC) CH27 APCs were fixed and stained with phalloidin at an early (1-2min) and late (2-5min) time point at the peak of lamellal signaling and thereafter, respectively (Fig 2A and 2B, see methods for precise time point definition). Early, the entire T cell:APC interface displayed deep F-actin with the phalloidin fluorescent intensity at 57±2%/40±3% (periphery/center) above cellular background at 1μm and 19±1%/11±1% at 2μm away from the interface (Fig 2C). Late, F-actin didn’t reach as deep into the T cell consistent with the diminished occurrence of the lamellal pattern (Fig 2C). Corroborating that actin reaches deep into the T cell in live cells, GFP-actin and the F-actin sensor F-tractin conjugated to GFP [15] displayed similar intensity distributions as a function of the distance from the interface (Fig 2C–2E and S3 Video). Actin reached further into the T cell than the TCR but not as far as lamellal signaling intermediates (Fig 1G). The extension of actin accumulation further away from the interface relative to the TCR is consistent with an actin matrix extending across the entire T cell:APC interface deep into the T cell. The even further extension into the T cell of the lamellal signaling intermediates is consistent with a scenario, where narrower cortical actin co-exists with the deep actin matrix with lamellal signaling preferentially associating with the latter. Alternatively, signaling intermediates accumulating the furthest from the interface may be not actin associated.

### The T cell:APC interface has a dynamically regulated undulating architecture

**Fig 3:**
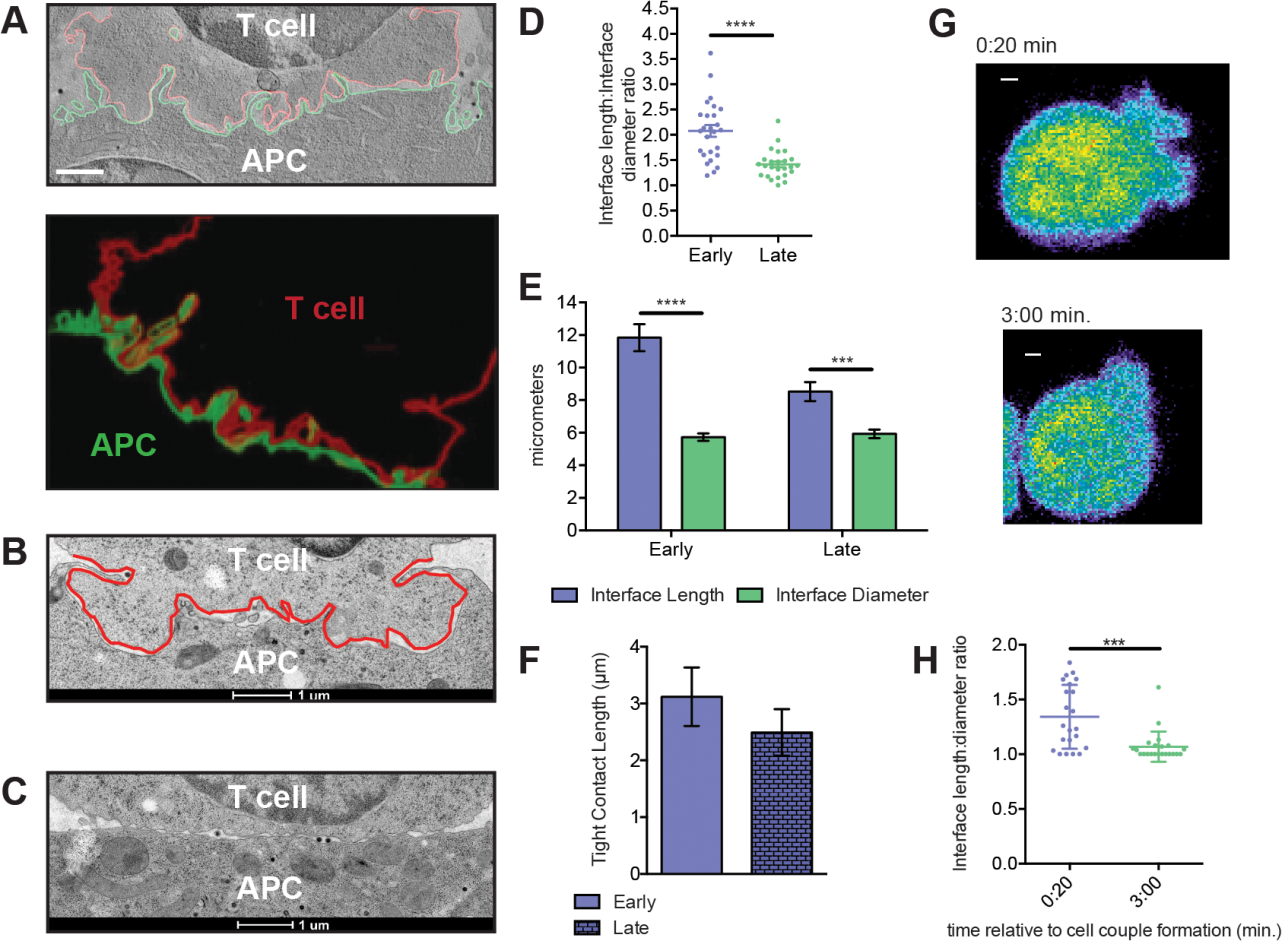
An undulating and highly interdigitated T cell:APC interface is dynamically regulated. (**A**) From an EM tomogram of an early (1-2min, see methods for precise time point definition) 5C.C7:CH27 APC (10μM MCC) interface (T cell in red, APC in green) a representative z-plane (top, from S4 Video) an a model of the entire reconstruction (bottom, from S5 Video) are given. (scale bar = 1μm). (**B**) A representative early (interface outlined in red) and (**C**) late electron micrograph of 5C.C7:CH27 APC interfaces (10μM MCC) are given. (**D**) The interface length to diameter ratio is given for early (number of cell couples analyzed across multiple independent experiments, n = 22) and late (n = 16) cell conjugates (see S1 Fig for analysis details)(see methods for precise time point definition). (**E**) The separate interface length and diameter measurements are given for the same cells as in D. Differences in interface length between the early and late time point are significant with p = 0.002. Differences in interface diameter are not significant. **(F)** Lengths of tight interface contact between at the T cell:APC (i.e. regions of opposing T cell:APC membrane <20nm apart rather than the entire interface outline denoted as ‘interface length’) are given from the same electron micrographs analyzed for D. **(G)** CFSE-labeled 5C.C7 T cell:CH27 APC interactions in the presence of 10 μM MCC peptide were imaged with an 100 x objective. Midplane sections of a representative T cell at 20s and 3min after tight cell coupling (as indicated) are given in a rainbow-like, false-color intensity scale (increasing from blue to red). The APC (not visible) is on the right (scale bar = 1μm). **(H)** The interface length to diameter ratios are given at the 20s and 3min time points for 22 cell couples. Error bars are s.e.m. Significance was determined by Student’s t-test (*p<0.05, ***p<0.001, ****p<0.0001).

T cell:APC interfaces are complex with considerable membrane curvature [16–19]. However, it is unknown how membrane topology is dynamically regulated during T cell activation. To relate membrane topology to the transient lamellal pattern, we delineated cell outlines at the 5C.C7 T cell:CH27 APC interface. Electron tomography revealed deep interface undulations within two minutes of tight cell coupling (Fig 3A, S4 and S5 Videos) that substantially increased the T cell surface area in close proximity to the APC. Quantifying this increase and determining the dynamics in single z-plane EM micrographs (Fig 3B and 3C), the undulating length of T cell membrane at the APC interface was 2.1±0.2 fold longer than the straight interface diameter early (1- 2min) and only 1.5±0.1 fold longer late (2-5min) (p<0.05, Fig 3D and 3E, see methods for precise time point definition), thus matching lamellal dynamics. Extrapolating the single z-plane data to the full cellular interface, the early 2.1-fold length increase corresponds to a dramatic ~ 4.5-fold increase in the area of T cell plasma membrane in close proximity to the APC. Interestingly, the length of tight contact, i.e. the part of the entire interface length with an inter-membrane distance that TCR/MHC-sized receptors/ligand couples can span, was comparable early and late (Fig 3F) as larger early interface length was balanced by a smaller fraction of tight contact. Large early membrane undulations thus may facilitate receptor ligand scanning but are unlikely to increase receptor ligand binding.

To determine whether the dynamic regulation of membrane undulations can also be observed in live cells and providing precise single cell temporal relations, we measured the interface length to diameter ratios in live CFSE-labeled T cells imaged with a 100x objective. Similar to the EM data, the interface length to diameter ratio was significantly greater at the early time point (1.35±0.05 at 20s after tight cell coupling versus 1.05±0.05 at 3min, p<0.001) (Fig 3G and 3H) even though absolute values were reduced because of inferior resolution.

To determine whether geometrical features of F-actin distributions are comparable to those of the membrane undulations, we further analyzed the fixed 5C.C7:CH27 conjugates stained with phalloidin and imaged by STED microscopy. Discreet regions of F-actin staining were oriented perpendicular to the interface plane early, as opposed to a more diffuse distribution late (Fig 2F and 2G). The depth to width ratio of resolved (135% of cell background) F-actin regions was 2.6±0.3 early compared to 0.7±0.1 late (p<0.0001, Fig 2F). The intensity to area ratio of the F-actin regions was 7.5±0.8 early as opposed to 1.2±0.1 late (p<0.0001, Fig 2H). The preferential orientation of discreet actin regions perpendicular to the interface during early T cell activation should contribute to an undulating and highly interdigitated T cell:APC interface by generating localized membrane projections that deform the T cell together with bound APC membrane. More diffuse later actin distributions align with the interface plane, favoring a flatter, more continuous geometry.

Thus we have characterized that actin and frequent membrane undulations reaching deep into the T cell across the entire T cell:APC interface are most prominent within the first few minutes to T cell activation.

### The lamellum associates with a large and diverse signaling network

**Fig 4:**
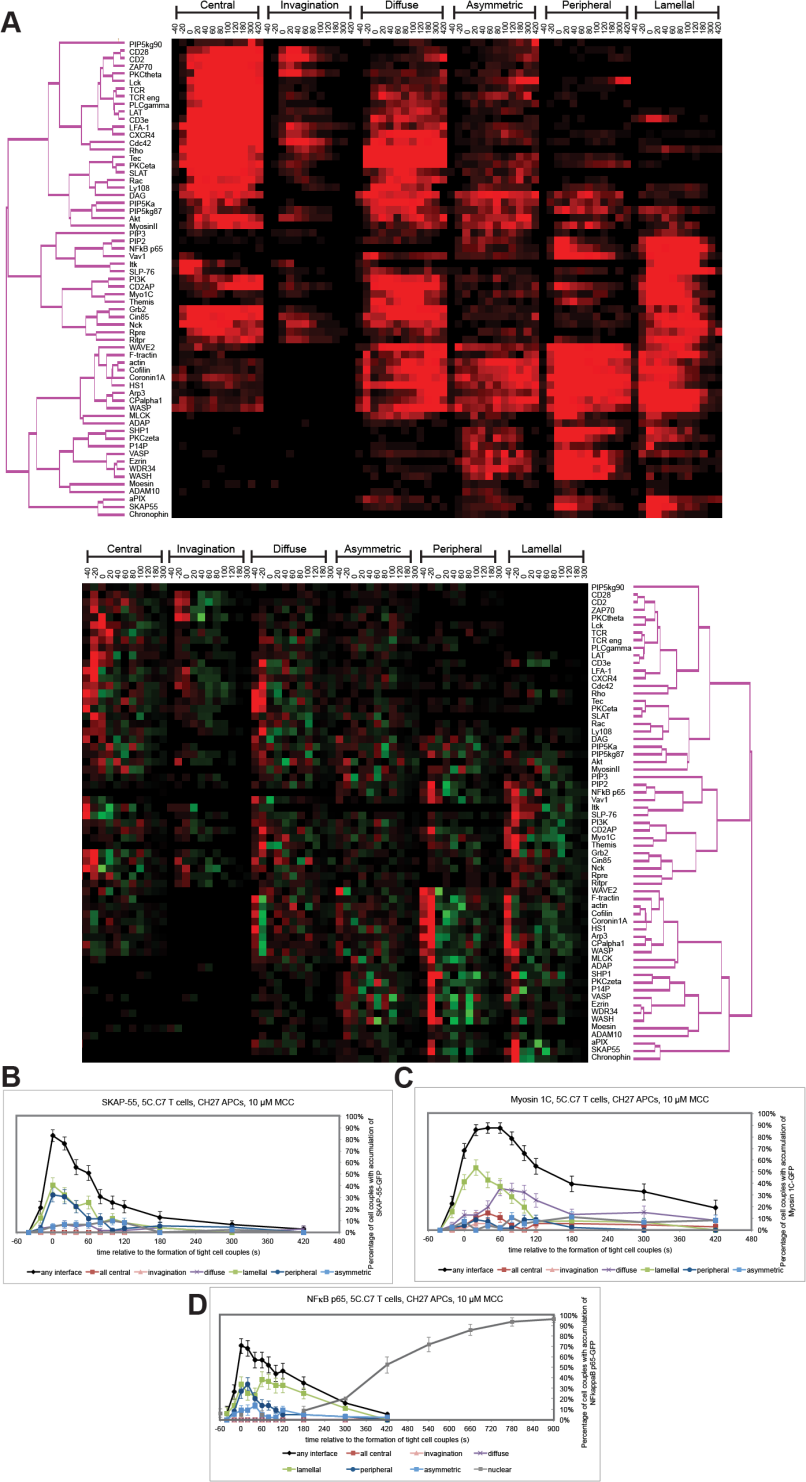
A large part of T cell signaling associates with the lamellum. (**A**) 5C.C7 T cells expressing the indicated sensors were activated on peptide loaded CH27 APCs (10μM MCC) and percentage occurrence of each pattern of interface enrichment (Fig 1A)[3] among all cell couples analysed across multiple experiments is given in shades of red from -40 to 420 s. In addition, to address the rate of pattern change, the percentage change per 20-s interval was tabulated (C-40 to L300 in the bottom part of the figure). Red indicates an increase and green a decrease in the percentage occurrence of a pattern relative to the previous time point. The cluster tree is given in pink. The source data for this figure and the sensors used are listed in S1 Table. **(B-D)** Pattern classification graphs, similar to Fig 1E, are given for (B) GFP-SKAP55 (number of cell couples analyzed across multiple independent experiments, n = 59), (C) Myosin 1C-GFP (n = 56), (D) NFκB p65-GFP (n = 44, % cell couples with nuclear accumulation of NFκB are given in addition in grey). Error bars are s.e.m.

To further investigate relations between the actin-associated lamellum and T cell signaling, we determined which signaling intermediates display significant lamellal patterning. In addition to SLP-76, PIP_2_, and Vav (Fig 1B–1F)(Figure 3C in [10]) they include e.g. Src-kinase associated phosphoprotein of 55 kDa (SKAP55), α-Pix, Myosin 1C, Themis [20], and nuclear factor κB (NFκB) prior to nuclear translocation (Fig 4 and S2H Fig). Out of the 54 signaling intermediates in our system data (the entire data minus receptors and actin) 20, i.e. 37%, showed lamellal patterning as the dominant pattern on at least one time point with 28–60% of cell couples showing lamellal patterning at that time (Fig 4A). Analyzing signaling distributions in DO11.10 T cell:A20 B cell lymphoma cell conjugates (S2O–S2Q Fig) for corroboration with a subset of 21 sensors, we found that SLP-76, PIP_2_, Vav, α-Pix and Myosin 1C displayed prominent lamellal localization as in 5C.C7 T cells. Lamellal localization was thus a widespread and general feature of T cell signaling. As discussed later, lamellal signaling is closely connected to signaling at the center of the T cell:APC interface. In the further characterization of the lamellal pattern we therefore frequently refer to the central one in comparison.

**Fig 5:**
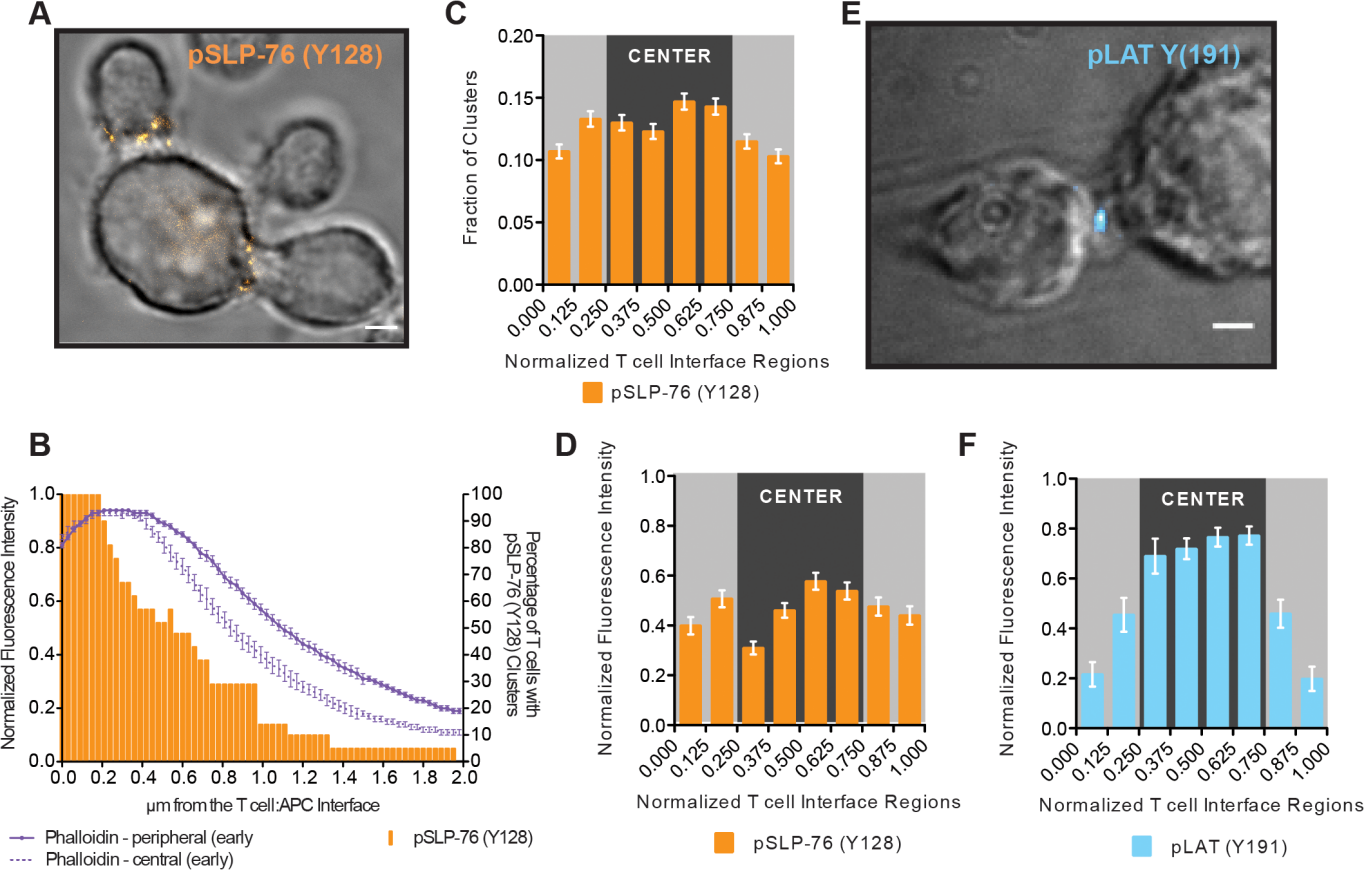
The active forms of the signaling intermediates SLP-76 and LAT display lamellal and central localization, respectively. (**A**) A representative STED image is given of two 5C.C7 T cells conjugated to a CH27 APC (10μM MCC) stained for pSLP-76 (Y128) at a single central z-plane as a DIC and fluorescence overlay (scale bar = 2μm). (**B**) F-actin amounts as a function of the distance from the interface measured from STED images are given for the early (<2min.) time point as in Fig 2C and plotted on the left y-axis (number of cell couples analyzed across multiple independent experiments, n = 26)(see methods for precise time points definition). The percentage of 5C.C7:CH27 conjugates (10μM MCC) imaged by STED with pSLP-76 (Y128) clusters is shown as a function of distance from the interface and plotted on the right y-axis (n = 21) (data from single color stains). (**C**) Individual pSLP-76 (Y128) clusters were identified and the fraction of these pSLP-76 clusters in each normalized interface region (interface diameter = 1, interface diameter divided into eight equal size sections) across the T cell:APC interface diameter from the same cells as in B is given. (**D**) To analyze pSLP-76 irrespective of clustering, the entire intensity distribution of pSLP-76 (Y128) is binned into normalized interface regions (interface diameter = 1, interface diameter divided into eight equal size sections) for the same cells as in B. (**E**) 5C.C7:CH27 conjugates (10μM MCC) stained for pLAT (Y191) were imaged by deconvolution microscopy and a representative image is given. (**F**) The intensity of pLAT (Y191) is given as in D (n = 12). Error bars are s.e.m.

**Fig 6:**
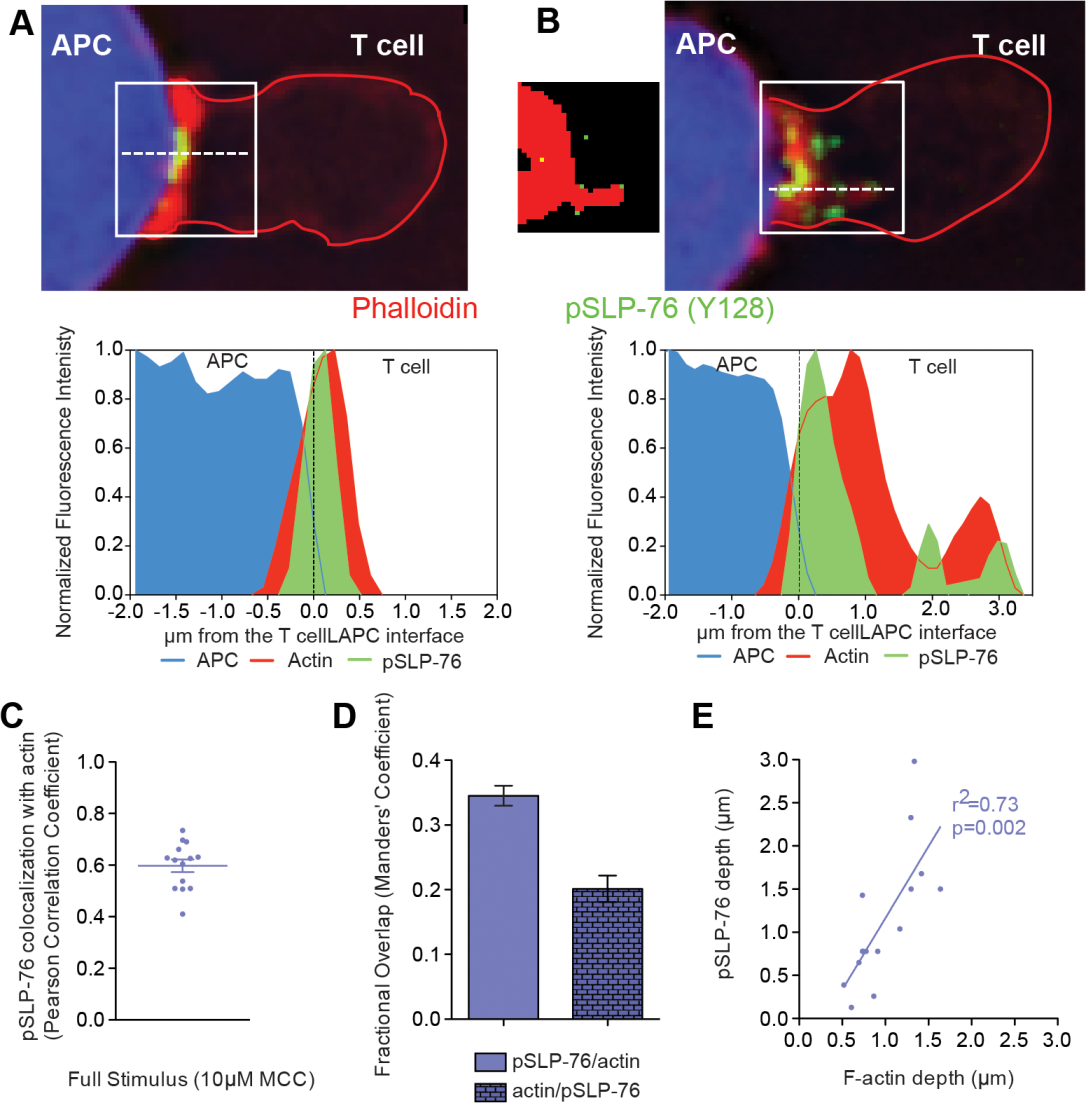
Activated SLP-76 localizes to lamellal F-actin structures. (**A, B**) Fixed 5C.C7 T cell:CH27 APC conjugates were stained for F-actin (Phalloidin, shown in red), pSLP-76 (Y128, shown in green), and APCs with Cell Trace Violet (shown in blue). The T cell outline is given in red. Representative images of (A) APC proximal and (B) lamellal localized pSLP-76 clusters are shown on top while intensity line scans perpendicular to the interface (dotted white line) are shown below with the black dashed line denoting the T cell:APC interface. A binary mask of above background F-actin (red) is shown with the centers of mass of pSLP-76 clusters (green) next to the representative image in panel B. (**C**) Pearson’s correlation coefficients for colocalization of pSLP-76 (Y128) with F-actin is given (number of cell couples analyzed across multiple independent experiments, n = 15). (**D**) Mander’s coefficient reporting the percentage of pSLP-76 (Y128) that overlaps with F-actin and vice versa was calculated for the same cell conjugates as in C. (**E**) The extent to which F-actin reaches into the T cell away from the interface was measured at the point of half-maximal fluorescence (average of peripheral and central measurements) and plotted against the distance of the deepest pSLP-76 (Y128) cluster from the interface with Pearson’s correlation coefficient for the same cell conjugates as in C. Error bars are s.e.m.

To investigate the extent of spatial overlap of lamellal signaling intermediates with actin, we addressed active signaling (Fig 5) and single cell relations (Fig 6). The distribution of phosphorylated SLP-76 Y128 (pSLP-76) in fixed cell couples was comparable to localization of SLP-76-GFP and actin, reaching from the T cell:APC interface deep into the cell across the entire width of the interface (Fig 5A–5D). Interestingly, fixed T cells showed distinct pSLP-76 clusters (Figs 5A and 6B) as discussed below. At the single cell level, as determined by staining fixed 5C.C7 T cell:CH27 conjugates for pSLP-76 and phalloidin (Fig 6A and 6B), pSLP-76 clusters colocalized with F-actin (average Pearson’s correlation coefficient = 0.6±0.1) (Fig 6C) and 34±2% of pSLP-76 was imbedded in F-actin (Fig 6D **)**. The extent to which pSLP-76 clusters and F-actin extend from the interface into the T cell correlated strongly (Pearson’s correlation coefficient = 0.73, p = 0.002) (Fig 6E). Addressing central signaling we corroborated that the previously established central localization of LAT-GFP **(** Fig 4A **)** [3] indeed represents active LAT by imaging of fixed cell couples stained for phosphorylated LAT Y191 (pLAT) (Fig 5E and 5F).

Lamellal signaling intermediates represent a third of the T cell signaling network components studied here and substantially overlap with the transient deep actin matrix emanating from the undulating T cell:APC interface. As discussed in more detail below, two mechanistic scenarios are conceivable. Signaling intermediates/complexes could bind directly to lamellal actin or actin could function as a three-dimensional lattice to trap signaling complexes. Either way, restraining signaling intermediates in the lamellal actin matrix should enhance the efficiency of lamellal signaling interactions.

### Lamellal signaling intermediates and actin diffuse similarly

**Fig 7:**
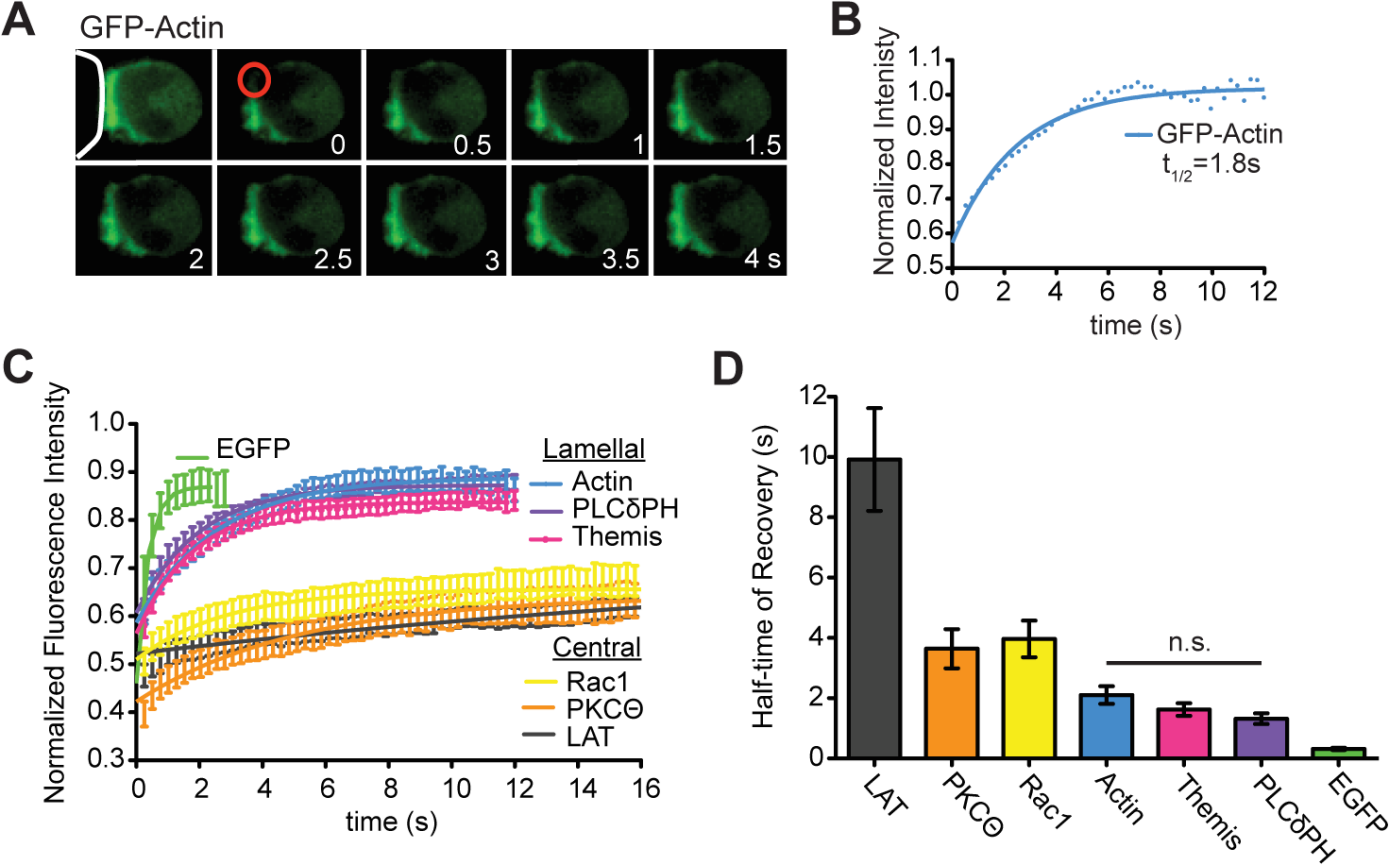
Actin and lamellal signaling intermediates localize and move comparably. (**A**) A fluorescence recovery after photobleaching (FRAP) time series is shown for GFP-actin with an initial prebleached image and post-bleached images spaced 255ms (APC is represented by white line, images shown in 0.5s intervals, bleach spot = red circle). (**B**) The corresponding fitted recovery curve is given. (**C**) Average fitted recovery curves for GFP fusions with the lamellal localized signaling intermediates PLCδPH (number of cell couples analyzed across multiple independent experiments, n = 11) and Themis (n = 18) and centrally localized PKCθ (n = 9), LAT (n = 10), an active Rac sensor [3](n = 13) plotted with GFP-actin (n = 10) and GFP (n = 10) control are shown. (**D**) The average half times of recovery for the same cell conjugates analyzed for C. (the line indicates significance by 1 way ANOVA, p>0.05). Error bars are s.e.m.

To further investigate the relation between lamellal signaling localization and actin, we determined whether lamellal signaling intermediates and actin diffused similarly as to be expected if lamellal signaling intermediates were bound to or trapped by the lamellal actin matrix. In fluorescence recovery after photobleaching (FRAP) experiments with Themis-GFP or PLCδPH-GFP the average half times of recovery of actin and the lamellal signaling intermediates were indistinguishable ranging from 1.3±0.2s for PLCδPH to 2.1±0.3s for actin (p>0.05) (Fig 7). GFP as a freely diffusible protein not involved in signaling recovered more rapidly (t_1/2_ = 0.32±0.04, p<0.0001) (Fig 7C and 7D). Signaling intermediates that accumulate at the center of the T cell:APC interface (PKCθ, LAT, and activated Rac1) showed substantially less recovery (30±2% to 44±1%) than lamellal proteins (63±2% to 76±2%) (Fig 7C) with significantly increased recovery half times in the mobile fraction of > 3.5s (p≤0.05 versus actin) **(** Fig 7D), suggesting a distinct, less mobile signaling structure. Importantly, the comparable μm-scale mobility of actin and lamellal signaling intermediates is consistent with both suggested mechanisms of actin-mediated lamellal signaling localization, that actin directly binds signaling intermediates such that they move together or that actin traps unbound signaling intermediates and thus slows their motion.

### The lamellum is an integrated component of the spatiotemporal organization of T cell signaling

**Fig 8:**
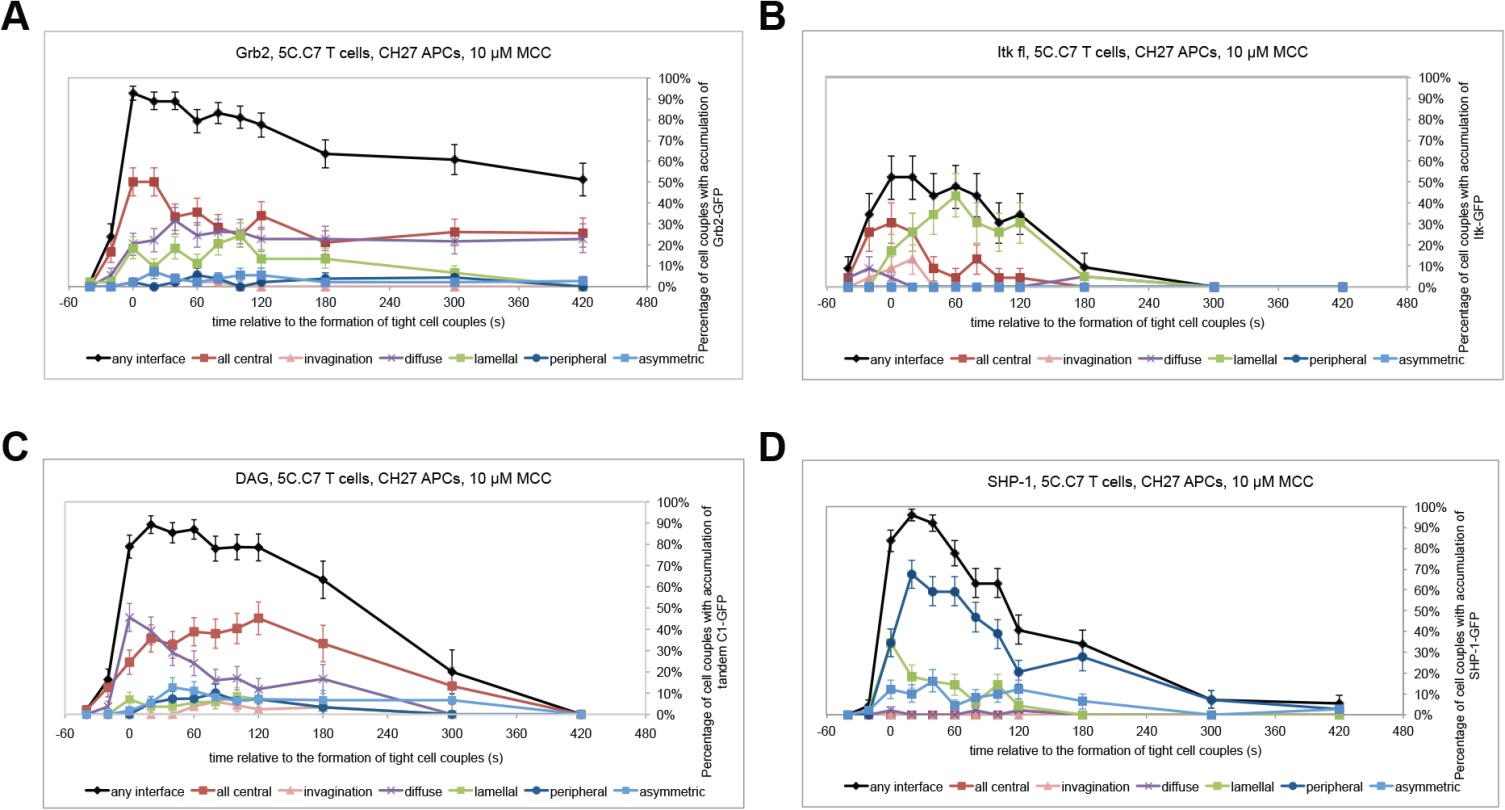
The lamellum is an integrated component of the spatiotemporal organization of T cell signaling. Pattern classification graphs, similar to Fig 1E, are given for (A) Grb2-GFP (number of cell couples analyzed across multiple independent experiments, n = 54), (B) Itk-GFP (n = 23), (C) the tandem C1 domain of PKCθ-GFP (Diacylglycerol (DAG) sensor) (n = 56) (late time points are not analyzable because of close apposition of Golgi-localized DAG to the interface), (D) SHP1-GFP (n = 49). Error bars are s.e.m.

For a system understanding of signaling organization in the activation of primary T cells by APCs we analyzed the patterning of additional of the more than 60 signaling intermediates covered (Figs 1, 4, 8 and S2 Fig). To help interpret these data it is useful to remember that the patterns analyzed in addition to the lamellal one also represent underlying cell biological structures. These structures include a central signaling complex [1, 3, 5, 21], a transient invagination [11], cortical accumulation and enrichment at the interface periphery as associated with a second actin-based structure, the peripheral actin ring [1, 14, 22–24]. Many proximal signaling intermediates (e.g. TCR, CD28, CD2, Lck, ZAP-70, LAT [3, 5]) were enriched at the interface center under inclusion of signaling intermediates in their active state (e.g. pLAT (Fig 5F) and active Rac [3]). A group of three adaptor proteins, SLP-76, Grb2, and Nck, dynamically connected central signaling to the lamellum while retaining some central enrichment to a variable extent, as again corroborated in DO11.10 T cell:A20 B cell lymphoma cell conjugates (S2O and S2Q Fig). Whereas SLP-76 after its peak enrichment at the interface center at the time of tight cell coupling moved completely to lamellal accumulation within one minute (Fig 1E), the transition of Nck and Grb2 to the lamellum was partial (Fig 8A)(Figure 3D in [10]). In particular Grb2 maintained substantial central enrichment over minutes (Fig 8A). The central to lamellal transition of the adaptor proteins was accompanied by that of the kinase Itk (Fig 8B). Thus enzymatic activity was potentially moved to the lamellum. The signaling intermediates subsequently enriched at the center and lamellum were largely distinct. The interface center contained signaling intermediates associated with core PKC signaling (e.g. DAG (Fig 8C), PKCθ[3]). Lamellal signaling intermediates, often showing a smaller extent of parallel peripheral accumulation, covered multiple signaling processes focused on the control of signal strength, including actin regulation by Vav1 and Myosin 1C (Fig 4C)(Figure 3C in [10]), signal attenuation by Themis [20], integrin avidity regulation by SKAP55 (Fig 4B). NFκB as a downstream signaling event was also enriched in the lamellum prior to nuclear entry (Fig 4D). Interestingly, many of these signaling intermediates have been linked to the three adaptor proteins SLP-76, Nck, and Grb2, suggesting that the adaptors are functional in the lamellum. Upon dissolution of the lamellum some of the lamellal signaling intermediates relocated to the central signaling complex, such as PI 3-kinase and CD2AP (Figure 3F in [10]), thus establishing a bi-directional feedback between central and lamellal signaling. Interestingly, the inhibitory signaling intermediate SHP-1 remained largely absent from both central and lamellal patterns, being mostly constrained to the periphery (Fig 8D). Such spatial sequestration of inhibitory signaling may favor early signal amplification.

## Discussion

A large-scale investigation of signaling in T cells activated by APCs revealed a complex and diverse spatiotemporal organization (Fig 4A)(Figure 3 in [10]). Because of the large number of signaling intermediates covered, these data should serve as a useful resource to understand associations between different elements of T cell signaling as they are governed by underlying cell biological structures. As prominent features, the interface center was enriched with proximal elements of T cell signaling (e.g. the TCR, CD28, Lck, ZAP-70, LAT, and pLAT), as well as with core signaling focusing on the PKC pathway (e.g. DAG and PKCθ)[1–3]. A lamellal distribution contained more distal signaling intermediates often involved in signal amplification (e.g. SLP-76, Vav, SKAP55, and Themis). Adaptor proteins such as SLP-76, Grb2, and Nck and signaling intermediates such as PI3K and CD2AP provided a bi-directional connection by moving from the center to the lamellum over time and vice versa. This organization differs dramatically from signaling organization in T cells activated by planar APC substitutes, where TCR signaling is initiated in peripheral microclusters that then move in the interface plane toward the center where signaling ceases [7, 25, 26].

The characterization of cellular structures underlying signaling distributions is critical for understanding signaling organization. Here we have characterized a large transient lamellum in detail that is supported by a F-actin network extending from an undulating and interdigitated T cell:APC interface several micrometers deep into the T cell. This structure was embedded into the larger spatiotemporal organization of T cells; lamellal actin occurred concurrent with peripheral and cortical actin distributions and lamellal signaling intermediates often displayed parallel or sequential accumulation in other patterns. Nevertheless, there was substantial distinction, as a number of signaling intermediates, such as PIP_2,_ α-Pix, or Chronophin (Fig 1F, S2D and S2H Fig), displayed almost exclusive lamellal localization. The large group of signaling intermediates extensively associated with the lamellal actin matrix, a good third of all covered here, displayed substantial overlap with the spatial, temporal, and μm-scale mobility features of the F-actin network. In an accompanying manuscript we have in addition established that modest interference with actin dynamics results in a parallel preferential loss of lamellal actin and lamellal signaling localization and activity. In combination these data strongly support the notion of the lamellal actin matrix as a distinct structure that is nevertheless well connected to other elements of the overall spatiotemporal organization of the T cell. Distinguishing molecular determinants of this structure need to be determined in the future. An earlier described concurrent large T cell invagination [11] is a different structure characterized by obligatory central localization and a distinct set of associated signaling intermediates (Fig 4A). The relation to ‘invadosomes’ [17] is possibly closer but unresolved.

Lamellal signaling localization could be mediated by direct binding of lamellal signaling intermediates to actin. For example ADAP physically links the SLP-76 signalosome to F-actin via VASP [27]. Alternatively, F-actin could function as a scaffold trapping signaling complexes. Such complexes could be nucleated in the central region of preferential signal initiation and subsequently diffuse to the lamellum as seen e.g. for SLP-76 (Fig 1E). Alternatively, lamellal signaling complexes could be generated by the direct engagement of less dense receptors in the lamellal region. Each of the different models of lamellal signaling complex generation and localization, as to be further investigated, is compatible with our data.

Molecule behavior in the lamellum was distinct from that of the center. Lamellal accumulation was transient and enriched molecules were mobile, consistent with small and dynamic signaling assemblies. The precise size range of lamellal signaling assemblies is difficult to resolve. Fixed cell staining for active pSLP-76 revealed distinct punctate structures implicating larger clusters, while in live cell imaging, total SLP-76 was distributed evenly across the lamellum, implicating smaller ones. As we have previously observed punctate structures comparable to the pSLP-76 clusters by live cell imaging (e.g. Arf6 sensor in [3]) detergent extraction may limit fixed cell data to larger clusters by removing smaller ones. In contrast to lamellal localization, central accumulation was sustained over minutes if not more and enriched molecules were close to immobile, suggesting a stable and highly cross-linked micrometer scale protein assembly [28]. While central and lamellal accumulation were partially overlapping and dynamically connected, the existence of two cell biologically distinct regions of T cell signaling suggests that they may serve different functions.

T cells must detect low doses of antigen with only modest distinction from self. Stable central signaling even in response to weak stimuli, as suggested by effective central PKCθ accumulation at limiting peptide concentrations [3], may provide a robust hub for sensitive core signaling. The lamellum with smaller more dynamic complexes is well suited to amplify early signaling. In this scenario, engagement of the TCR together with costimulatory receptors would drive transient actin dynamics [29] required for lamellal signaling as further explored in an accompanying manuscript. Being enriched in both actin regulators and signaling intermediates, lamellal signaling complexes could then provide positive feedback to amplify T cell signaling at its peak [29, 30].

Beyond amplifying signaling by constraining a large part of the T cell signaling system in an actin matrix, the lamellum with its associated membrane undulations provides additional potential mechanisms to shape T cell activation. The more than four-fold increase in T cell:APC contact area resulting from the early membrane undulations should enhance ligand scanning. Altered membrane curvature and the dramatically increased membrane surface to cytoplasm volume ratio in T cell membrane extensions should alter signal progression [31–33]. Plasma membrane undulations are highly deformed membrane structures that could affect molecular interactions at the membrane as Bin/Amphiphysin/Rvs (BAR) proteins maintain curved membrane structures and provide a link to various actin regulators [34]. The early T cell lamellum likely constrains actin-mediated transport. While our fluorescence microscopy studies only visualize gross actin topology and not the orientation of individual actin filaments, this gross topology strongly suggests that retrograde actin flow should be directed towards the base of T cell invaginations. Moreover, invaginations should obstruct interface-wide continuous F-actin assemblies and thus impede cortical movement towards the interface center on the scale of several μm. Thus early signaling clusters are likely to become trapped across the lamellum in a range of small sizes. Once the lamellal pattern has mostly dissolved, a flatter interface topology should be more conducive to long-range lateral transport required to build and/or sustain larger protein clusters [1, 35]. It is unclear how the early preferential actin orientation perpendicular to the cellular interface can be related to actin in T cells activated on planar APC substitutes [22–24]. As bilayers cannot be deformed outward-pointing actin structures are unlikely to develop into membrane protrusions and may be blocked or diverted sideways. This difference likely drives a divergent subcellular signaling organization with an extensive formation and motion of microclusters less likely in T cell:APC couples.

In summary, as part of a large-scale investigation of signaling organization in primary T cells as activated by APCs we have characterized a transient F-actin matrix extending from an undulating and highly interdigitated T cell:APC interface several micrometers into the T cell that is associated with a large part of the signaling system at the peak of T cell signaling activity. Similar actin sheets with embedded signaling complexes linked to undulating cellular interfaces should play a comparably important role whenever dynamic cell contacts drive cell fate decisions, such as in development [36], stem differentiation [37], and cancer metastasis [38]. Many of the signaling intermediates enriched in the lamellal pattern in T cells are critical regulators of these processes [39, 40].

## Materials and Methods

### Antibodies and Reagents

Antibodies for fixed cell microscopy were Alexa 488-α-SLP76 pY128 (BD Pharmingen) and α-LAT pY191 (Cell Signaling) with Alexa 488-goat α-rabbit IgG (Invitrogen). F-actin was stained with Alexa 633 or 488-Phalloidin (Invitrogen). CFSE and Cell Trace Violet were used as whole cell stains (Invitrogen).

### Mice and Cells

*In vitro* primed primary 5C.C7 T cells were set up from preferentially female 5C.C7 TCR transgenic mice of about 2 months of age, as previously described [3]. The use of all mice has been reviewed and approved by the UT Southwestern IACUC committee and is covered by a University of Bristol Home Office license, respectively.

### Microscopy and Image Analysis

All sensors used are given in S1 Table. As previously described in detail [3], T cells were transduced with MMLV-based retroviral particles to allow fluorescent sensor expression, transduced T cells were sorted for low GFP expression (2.6μM±0.4) to maximize physiological significance, and the interaction of sorted T cells with CH27 B cell lymphoma APCs loaded with 10μM moth cytochrome C peptide (fragment 88–103) was imaged at 37°C. Every 20 seconds a differential interference contrast image and 21 fluorescence z-planes spaced 1μm (total z volume = 20μm) were acquired with a CoolSnap HQ2 camera (Photometrics) and Metamorph (Molecular Devices) using a 40x (NA = 1.3) oil objective. Patterns of signaling sensor enrichment were assessed according to previously established quantitative criteria (Figure 2 in [3]). Briefly, the six, mutually exclusive interface patterns were: accumulation at the center of the T cell-APC interface (central), accumulation in a large T cell invagination (invagination), accumulation that covered the cell cortex across central and peripheral regions (diffuse), accumulation in a broad interface lamellum (lamellum), accumulation at the periphery of the interface (peripheral) or in smaller protrusions (asymmetric). Briefly, for each cell couple at each time point we first determined whether fluorescence intensity in the area of accumulation was >40% above the cellular fluorescence background. If so, the geometrical features of the area of accumulation, fraction of the interface covered, location within the interface, and extension of the area of accumulation away from the interface (Figure 2 in [3]), were used to assign the cell couple to one of the mutually exclusive patterns. Systems-scale cluster analysis was performed with Cluster (Michael Eisen, UC Berkeley) as established [3].

For fixed cell imaging, CH27 APCs were first adhered to a poly-L-lysine coated coverslip. T cells were then allowed to interact with APCs for 2 or 7min for early or late time points, respectively. T cells were fixed with 4% EM grade paraformaldehyde in PBS at 4°C for 20min and then stained for stimulated-emission depletion (STED) microscopy or deconvolution microscopy and mounted with ProLong Antifade (Invitrogen). For STED microscopy, T cells were stained with Alexa-488 conjugated Phalloidin or Alexa-488 conjugated α-pSLP-76 (Y128) and imaged as previously described in detail [12]. Briefly, cells were imaged through a 100×1.4 NA HCX APO objective on a Leica TCS STED CW system controlled by Leica AS AF software. Alexa Fluor 488 was excited using a 488nm Argon laser and STED depletion was achieved using a 592nm continuous wave fiber laser. For deconvolution microscopy in up to three colors a pDV Deltavision microscope (Applied Precision) equipped with an Olympus APO 40x oil objective (NA = 1.3) and Cool Snap HQ2 camera (Photometrics) was used. Image acquisition and deconvolution with a constrained iterative deconvolution algorithm were performed with softWoRx software v 2.0 (Applied Precision). A single DIC reference image and fluorescent z-stacks spanning the entire cell (0.2μm z-step) were acquired for each field. All image analysis for fixed cell microscopy was performed in Image J (NIH) as described below.

In the fixed cell couple experiments the experimental timing of cell coupling by control of cell contact duration needed to be complemented by morphological filtering during analysis, as the shape of some T cells in the ‘late’ samples made it apparent that some cell couples had formed only briefly before fixation. We used two morphological criteria for the post acquisition identification of ‘early’ cell couples, the presence of a uropod and T cell elongation. The T cell uropod is largely restricted to the first minute of cell coupling (S1 Fig). Similarly, as determined by live cell imaging the ratio of T cell length (distance from the interface to the posterior end of the cell) to T cell diameter (widest part of the T cell parallel to the interface) was 1.35±0.05 at the time of cell coupling and plateaued at 1.2±0.05 between 2 and 5min after cell coupling (p<0.05). Therefore, T cells with a distinct uropod or a cell length to diameter ratio of >1.25 were considered ‘early’. Measurements of how deep actin and lamellal localized signaling intermediates reach into the T cell away from the interface were calculated using the box tool in Image J, either with a single box spanning the entire interface or with separate boxes for the interface center (inner 50% of the interface diameter) and periphery (outer 25%). The first box was at the interface, equal size boxes were then moved into the T cell in defined distance increments. Colocalization analysis for pSLP-76 and F-actin was performed with the JACoP plugin for Image J. Briefly, a binary mask for each channel was generated by linear thresholding and colocalization was assessed by calculation of the Pearson’s correlation coefficient (PCC) and Mander’s overlap coefficient. The distance of phosphorylated SLP-76 clusters from the interface was assessed by line scans and calculated from the point at which the APC fluorescence dropped to half-maximum. Phosphorylated SLP-76 clusters were evaluated with the Object Counter 3D plugin for Image J.

To visualize membrane undulations by live cell imaging, T cells were loaded with 2 μM CFSE for 15min at 37°C and their interaction with CH27 APCs and 10 μM MCC peptide was imaged with a 100 x objective (NA = 1.4) on an Perkin Elmer UltraVIEW ERS 6FE spinning disk confocal microscope at 37°C. Every 20 seconds a differential interference contrast image and 41 fluorescence z-planes spaced 0.4μm (total z volume = 16μm) were acquired. Interface length and diameter were measured in midplane sections as in the EM images (as described in S1 Fig).

### Electron Microscopy

5C.C7 T cells and peptide-loaded CH27s (10μM MCC) were centrifuged together for 30s at 350g to synchronize cell coupling, the cell pellet was immediately resuspended to minimize unspecific cell coupling and cellular deformation, and the cell suspension was further incubated at 37°C. After 2 and 5min for early and late time points, respectively, the cell suspension was high pressure frozen and freeze substituted to Epon as described previously [41]. Briefly, the Leica EM PACT2 with a Rapid Transfer System was used to high-pressure freeze T cell:APC suspensions. Frozen samples were substituted with 1%osmium tetroxide plus 0.1%uranyl acetate in acetone at -90°C, and subsequently embedded in Epon. Ultrathin sections were analyzed in a FEI Tecnai12 Biotwin equipped with a bottom-mount 4*4K EAGLE CCD camera. T cell:APC couples were identified in electron micrographs through their wide cellular interface. As described above, the time point assignment of cell couples was filtered with morphological criteria post acquisition using presence of a uropod and T cell elongation. In addition, cell couples with a distance of the nucleus from the cellular interface of more than 1μm were classified as early, as this distance decreased from 1.4±0.1μm in the early samples to 0.7±0.1μm (p<0.001) late. Fulfillment of two of the three criteria was sufficient for time point assignment. The extent of interface undulations was analyzed as described in S1 Fig. A tomography data series was acquired in a FEI Tecnai 20 TEM between -70° and +70° with a 1.5° increment [18]. The data was reconstructed using IMOD etomo software [16]. Segmentation was made using AMIRA software (VSG), while for visualization, a combination of IMOD, AMIRA and Image J were used.

### Fluorescent Recovery after Photobleaching

Individual 5C.C7 T cell:CH27 APC conjugates were focused on and bleaching was done within the first 2min of cell conjugate formation. A pDVRT Deltavision deconvolution microscope (Applied Precision) equipped with a Quantitative laser module for FRAP, an Olympus APO 40x oil objective (NA = 1.3), and Cool Snap HQ2 camera (Photometrics) and controlled with Deltavision softWoRx software was used. All FRAP was performed at 37°C. Three prebleach images were acquired and then 10×10ms 488nm laser pulses (100% power) bleached a ~1μm Gaussian spot at the T cell:APC interface to near 50% of the prebleach intensity. Post bleach images were acquired every 255ms for a total of 30s to 2min depending on the protein. Analysis of recovery was performed manually in Image J by calculating the intensity in the bleach spot before and after bleaching. Background subtracted data was normalized to the average intensity of the 3 prebleached images and was fitted in Prism (Graphpad) with the equation Y_(f)_ = (Y_max_-Y_min_)(1-e^-kt^)+Y_min_. Y_(t)_ is the intensity of fluorescence at time t, Y_max_ and Y_min_ are the maximum and minimum intensities of fluorescence post-bleaching and k is the rate constant of recovery.

### Statistical Analysis

To determine a significant change in spatiotemporal patterning, a proportion z-test was performed. Otherwise, statistical significance was determined with an unpaired Student’s *t*-test or 1-WAY ANOVA when appropriate. Statistical analysis was performed with GraphPad Prism (v5.0) or in some cases Excel.

## Supporting Information

S1 Fig Image analysis. (A) Shown is a representative electron micrograph of the same 5C.C7 T cell:APC conjugate as in Fig 3B. The interface length is outlined (blue) and the interface diameter is drawn (red). The lines were drawn on images in Metamorph and the micrometer lengths were recorded and the ratio was calculated. (B) Shown is a representative STED image of a 5C.C7 T cell:APC conjugate stained with phalloidin, the same as in Fig 2A (APC outline in white, scale bar = 2μm). The number of F-actin structures (labeled 1–5) was determined by linear scaling and each structure was measured with linescans. One linescan was oriented perpendicular to the interface to measure the depth of the structure (red dotted line) and the other was oriented parallel to the interface to measure the width (red dashed line). The intensity profile was plotted and the depth and width measurements were made at the full-width half-maximum of the F-actin structure profile (see graphs below image). (C) Uropod retraction. 5C.C7 T cells were activated with CH27 APCs and 10 μM MCC agonist peptide. The percentage of cell couples with a visible uropod is given with standard errors relative to tight cell coupling. A T cell was scored to have a uropod as long as an inversion of curvature of the plasma membrane could be detected at the distal pole in the DIC images. 70 cell couples were analyzed.
(TIF)

S2 Fig A large-scale network of activated T cell signaling intermediates localize to the actin-rich T cell lamellum. (**A-N**) 5C.C7 T cells expressing the indicated sensors were activated on peptide loaded CH27s (10μM MCC) and percentage occurrence of each pattern of interface enrichment (Fig 1A)[3] among all cell couples analysed across multiple experiments is given in pattern classification graphs similar to Fig 1E. (A) ADAM10-GFP (number of cell couples analyzed across multiple independent experiments, n = 48), (B) ADAP-GFP (n = 43), (C) Akt-GFP (n = 45), (D) Chronophin-GFP (n = 54), (E) Ezrin-GFP (n = 52), (F) Moesin-GFP (n = 49), (G) Myosin light chain kinase-YFP (MLCK) (n = 32), (H) α-Pix-GFP [5] (n = 64), (I) PKC**ζ**-GFP (n = 48), (J) the negative charge sensor R-pre-GFP (n = 47), (K) GFP-SLAT [5] (n = 60), (L) GFP-VASP (n = 49), (M) GFP-WASH (n = 48), (N) WDR34-GFP (n = 58). Error bars are s.e.m. (**O**) DO11.10 T cells expressing the indicated sensors were activated on peptide loaded A20 B cell lymphoma APCs (10μM Ova) and patterns of interface enrichment were scored: Cluster analysis of the data presented is based on the six mutually exclusive interface patterns [central (C), invagination (Inv), diffuse (D), asymmetric (AC), peripheral (P), and lamellum (L), see Fig 1A] is given as described previously [3]. The percentage occurrence of each pattern is given in shades of red from C-40 to L420 in the top part of the figure. In addition, to address the rate of pattern change, the percentage change per 20-s interval was tabulated (C-40 to L300 in the bottom part of the figure). Red indicates an increase and green a decrease in the percentage occurrence of a pattern relative to the previous time point. (**P, Q**) The pattern classification data of many of the molecules included in the cluster analysis in panel R have been previously published. In panels P and Q new pattern classification graphs, similar to Fig 1E, are given: DO11.10 T cells expressing the DAG sensor (P, n = 47) or Nck-GFP (Q, n = 52) were activated on peptide loaded A20 B cell lymphoma APCs (10μM OVA) and the pattern classification graphs are given.
(TIF)

S1 Table Sensors, source pattern classification data, and representative videos for all signaling intermediates covered. For the signaling intermediates covered in Fig 4A sensors used, source pattern classification data, and representative videos are listed as figures and supplementary videos in this publication or as a prior publication. An asterisk indicates a sensor that hasn’t been published before. Names in parentheses indicate collaborators who have provided a plasmid containing the sensor. All data are also openly available on the Wuelfing laboratory website at the University of Bristol at http://www.bristol.ac.uk/cellmolmed/research/infect-immune/wuelfing/spatiotemporal-patterning/.
(DOCX)

S1 Video Representative interactions of 5C.C7 T cells retrovirally transduced to express the indicated sensors with CH27 B cell lymphoma APCs in the presence of MCC agonist peptide (10 M) are shown in S1 to S3 Videos. DIC images are shown on the top, with matching top-down, maximum projections of 3D sensor fluorescence data on the bottom. The sensor fluorescence intensity is displayed in a rainbow-like, false-color scale (increasing from blue to red). 20 s intervals in video acquisition are played back as 2 frames per second. The 5C.C7 T cell in S1 Video is transduced with SLP-76-GFP. Cell coupling occurs in frame 5 (2s indicated video time). A rapid transition from central to lamellal accumulation that subsequently fades more slowly is shown.
(MOV)

S2 Video The video is displayed similar to S1 Video. The 5C.C7 T cell in S2 Video is transduced with Vav1-GFP. Cell coupling occurs in frame 4 (1s indicated video time). A transient lamellal accumulation is shown.
(MOV)

S3 Video The video is displayed similar to S1 Video. The 5C.C7 T cell in S3 Video is transduced with F-Tractin-GFP. Cell coupling occurs in frame 6 (2s indicated video time). Accumulation in a mix of peripheral, lamellal, and asymmetric patterns is shown.
(MOV)

S4 Video A representative EM tomogram of a 5C.C7 T cell interacting with a CH27 B cell lymphoma APC in the presence of MCC agonist peptide (10 M) is shown in the S4 and S5 Videos. In S4 Video individual z-slices of the EM tomogram reconstruction with T and B cell membranes marked in red and green are shown.
(MOV)

S5 Video For the same EM tomogram as in S4 Video a model of the EM tomogram reconstruction with T and B cell membranes marked in red and green is shown. (MOV)

S6 Video The video is displayed similar to S1 Video. The 5C.C7 T cell in S6 video is transduced with ADAM10-GFP.
(MOV)

S7 Video The video is displayed similar to S1 Video. The 5C.C7 T cell in S7 video is transduced with GFP-ADAP.
(MOV)

S8 Video The video is displayed similar to S1 Video. The 5C.C7 T cell in S8 video is transduced with Akt-GFP.
(MOV)

S9 Video The video is displayed similar to S1 Video. The 5C.C7 T cell in S9 video is transduced with Chronophin-GFP.
(MOV)

S10 Video The video is displayed similar to S1 Video. The 5C.C7 T cell in S10 video is transduced with tandem C1-GFP, a sensor for DAG.
(MOV)

S11 Video The video is displayed similar to S1 Video. The 5C.C7 T cell in S11 video is transduced with Ezrin-GFP.
(MOV)

S12 Video The video is displayed similar to S1 Video. The 5C.C7 T cell in S12 video is transduced with Grb2-GFP.
(MOV)

S13 Video The video is displayed similar to S1 Video. The 5C.C7 T cell in S13 video is transduced with Itk-GFP.
(MOV)

S14 Video The video is displayed similar to S1 Video. The 5C.C7 T cell in S14 video is transduced with Lck-GFP.
(MOV)

S15 Video The video is displayed similar to S1 Video. The 5C.C7 T cell in S15 video is transduced with Moesin-GFP.
(MOV)

S16 Video The video is displayed similar to S1 Video. The 5C.C7 T cell in S16 video is transduced with MLCK-YFP-CaM-CFP.
(MOV)

S17 Video The video is displayed similar to S1 Video. The 5C.C7 T cell in S17 video is transduced with Myosin 1C-GFP.
(MOV)

S18 Video The video is displayed similar to S1 Video. The 5C.C7 T cell in S18 video is transduced with Nck-GFP.
(MOV)

S19 Video The video is displayed similar to S1 Video. The 5C.C7 T cell in S19 video is transduced with GFP-p65.
(MOV)

S20 Video The video is displayed similar to S1 Video. The 5C.C7 T cell in S20 video is transduced with GFP-α-Pix.
(MOV)

S21 Video The video is displayed similar to S1 Video. The 5C.C7 T cell in S21 video is transduced with PKCζ-GFP.
(MOV)

S22 Video The video is displayed similar to S1 Video. The 5C.C7 T cells in S22 video is transduced with GFP-R-pre, a sensor for negatively charged lipids.
(MOV)

S23 Video The video is displayed similar to S1 Video. The 5C.C7 T cell in S23 video is transduced with SHP-1-GFP.
(MOV)

S24 Video The video is displayed similar to S1 Video. The 5C.C7 T cell in S24 video is transduced with GFP-SLAT.
(MOV)

S25 Video The video is displayed similar to S1 Video. The 5C.C7 T cell in S25 video is transduced with GFP-VASP.
(MOV)

S26 Video The video is displayed similar to S1 Video. The 5C.C7 T cell in S26 video is transduced with GFP-WASH.
(MOV)

S27 Video The video is displayed similar to S1 Video. The 5C.C7 T cell in S27 video is transduced with GFP-WDR34-GFP.
(MOV)
