# Supplementary figures and images for: Early Signaling in Primary T Cells Activated by Antigen Presenting Cells Is Associated with a Deep and Transient Lamellal Actin Network
Source: PLoS One. 2015 Aug 3;10(8):e0133299. doi: 10.1371/journal.pone.0133299 (PMC4523204; doi:10.1371/journal.pone.0133299)

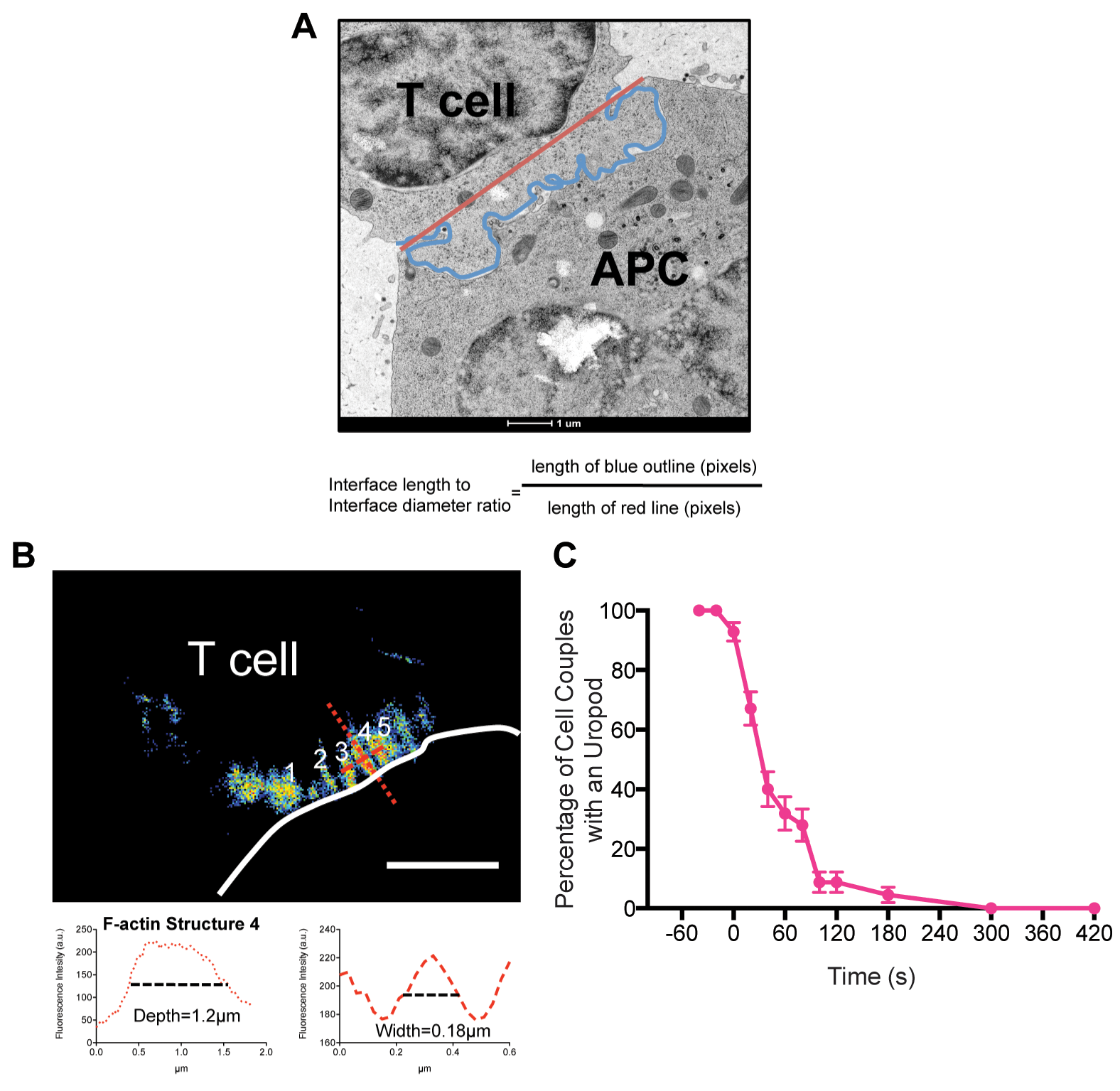

Supplement: S1 Fig — (A) Shown is a representative electron micrograph of the same 5C.C7 T cell:APC conjugate as in Fig 3B. The interface length is outlined (blue) and the interface diameter is drawn (red). The lines were drawn on images in Metamorph and the micrometer lengths were recorded and the ratio was calculated. (B) Shown is a representative STED image of a 5C.C7 T cell:APC conjugate stained with phalloidin, the same as in Fig 2A (APC outline in white, scale bar = 2μm). The number of F-actin structures (labeled 1–5) was determined by linear scaling and each structure was measured with linescans. One linescan was oriented perpendicular to the interface to measure the depth of the structure (red dotted line) and the other was oriented parallel to the interface to measure the width (red dashed line). The intensity profile was plotted and the depth and width measurements were made at the full-width half-maximum of the F-actin structure profile (see graphs below image). (C) Uropod retraction. 5C.C7 T cells were activated with CH27 APCs and 10 μM MCC agonist peptide. The percentage of cell couples with a visible uropod is given with standard errors relative to tight cell coupling. A T cell was scored to have a uropod as long as an inversion of curvature of the plasma membrane could be detected at the distal pole in the DIC images. 70 cell couples were analyzed. (TIF) [file pone.0133299.s001.tif]

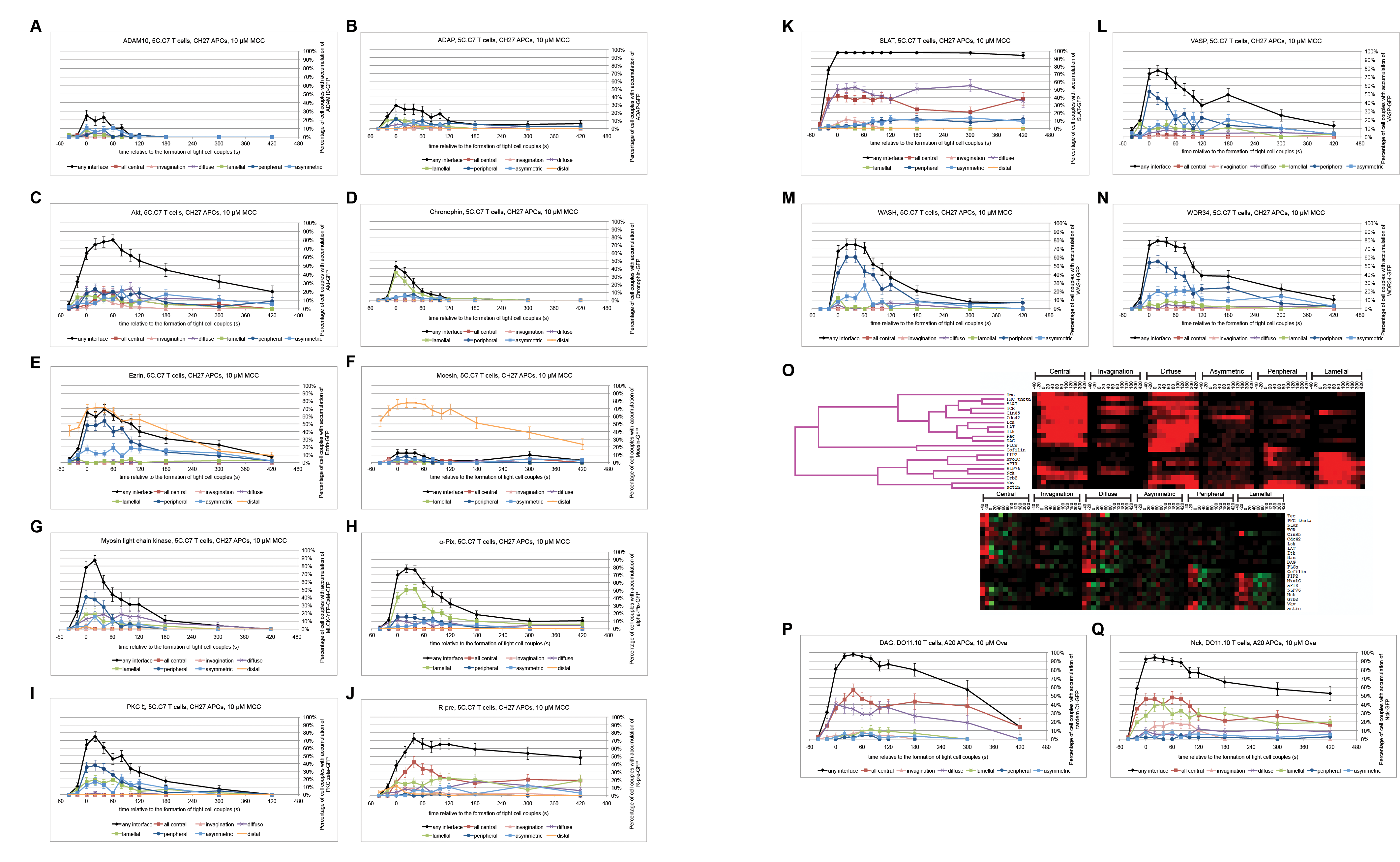

Supplement: S2 Fig — (A-N) 5C.C7 T cells expressing the indicated sensors were activated on peptide loaded CH27s (10μM MCC) and percentage occurrence of each pattern of interface enrichment (Fig 1A)[3] among all cell couples analysed across multiple experiments is given in pattern classification graphs similar to Fig 1E. (A) ADAM10-GFP (number of cell couples analyzed across multiple independent experiments, n = 48), (B) ADAP-GFP (n = 43), (C) Akt-GFP (n = 45), (D) Chronophin-GFP (n = 54), (E) Ezrin-GFP (n = 52), (F) Moesin-GFP (n = 49), (G) Myosin light chain kinase-YFP (MLCK) (n = 32), (H) α-Pix-GFP [5] (n = 64), (I) PKCζ-GFP (n = 48), (J) the negative charge sensor R-pre-GFP (n = 47), (K) GFP-SLAT [5] (n = 60), (L) GFP-VASP (n = 49), (M) GFP-WASH (n = 48), (N) WDR34-GFP (n = 58). Error bars are s.e.m. (O) DO11.10 T cells expressing the indicated sensors were activated on peptide loaded A20 B cell lymphoma APCs (10μM Ova) and patterns of interface enrichment were scored: Cluster analysis of the data presented is based on the six mutually exclusive interface patterns [central (C), invagination (Inv), diffuse (D), asymmetric (AC), peripheral (P), and lamellum (L), see Fig 1A] is given as described previously [3]. The percentage occurrence of each pattern is given in shades of red from C-40 to L420 in the top part of the figure. In addition, to address the rate of pattern change, the percentage change per 20-s interval was tabulated (C-40 to L300 in the bottom part of the figure). Red indicates an increase and green a decrease in the percentage occurrence of a pattern relative to the previous time point. (P, Q) The pattern classification data of many of the molecules included in the cluster analysis in panel R have been previously published. In panels P and Q new pattern classification graphs, similar to Fig 1E, are given: DO11.10 T cells expressing the DAG sensor (P, n = 47) or Nck-GFP (Q, n = 52) were activated on peptide loaded A20 B cell lymphoma APCs (10μM OVA) and the pattern class [file pone.0133299.s002.tif]
