# Supplementary material for: Early Signaling in Primary T Cells Activated by Antigen Presenting Cells Is Associated with a Deep and Transient Lamellal Actin Network
Source: PLoS One. 2015 Aug 3;10(8):e0133299. doi: 10.1371/journal.pone.0133299 (PMC4523204; doi:10.1371/journal.pone.0133299)
Supplement: S1 Table — For the signaling intermediates covered in Fig 4A sensors used, source pattern classification data, and representative videos are listed as figures and supplementary videos in this publication or as a prior publication. An asterisk indicates a sensor that hasn’t been published before. Names in parentheses indicate collaborators who have provided a plasmid containing the sensor. All data are also openly available on the Wuelfing laboratory website at the University of Bristol at http://www.bristol.ac.uk/cellmolmed/research/infect-immune/wuelfing/spatiotemporal-patterning/. (DOCX) [file pone.0133299.s003.docx]

**Table S1**

| **Signaling intermediate** | **Sensor** | **Location of classification data/representative video** |
| --- | --- | --- |
| Actin as GFP-Actin | GFP-Actin ([44](#_ENREF_44)) | ([45](#_ENREF_45)) |
| Actin as F-tractin | F-tractin-GFP ([25](#_ENREF_25)) | Fig. 2E, S3 video |
| ADAM10 | ADAM10-GFP * | S2A Fig., S6 video |
| ADAP | GFP-ADAP (C. Freund) | S2B Fig., S7 video |
| Akt | Akt-GFP * | S2C Fig., S8 video |
| Arp3 | Arp3-GFP * | Manuscript in preparation |
| Capping protein 1 α | Capping protein 1 α-GFP * | Manuscript in preparation |
| CD2 | CD48-GFP ([13](#_ENREF_13)) | ([3](#_ENREF_3)) |
| CD28 | B7-2-GFP ([23](#_ENREF_23)) | ([3](#_ENREF_3)) |
| CD2AP | GFP-CD2AP ([3](#_ENREF_3)) | ([12](#_ENREF_12)) |
| Cdc42 | WASP GBD-GFP-CAAX ([46](#_ENREF_46)) | ([3](#_ENREF_3)) |
| Chronophin | Chronophin-GFP * | S2D Fig., S9 video |
| Cin85 | GFP-Cin85 ([3](#_ENREF_3)) | ([3](#_ENREF_3)) |
| Cofilin | Cofilin-GFP ([5](#_ENREF_5)) | Manuscript in preparation |
| Coronin 1A | Coronin 1A-GFP * | Manuscript in preparation |
| CXCR4 | CXCR4-GFP ([3](#_ENREF_3)) | ([3](#_ENREF_3)) |
| DAG | Tandem C1-GFP ([47](#_ENREF_47)) (O. Acuto) | Fig. 8C, S10 video |
| Ezrin | Ezrin-GFP * | S2E Fig., S11 video |
| Grb2 | Grb2-GFP ([47](#_ENREF_47)) (M. Davis) | Fig. 8A, S12 video |
| HS1 | HS1-GFP * | Manuscript in preparation |
| Itk | Itk-GFP * | Fig. 8B, S13 video |
| LAT | LAT-GFP ([3](#_ENREF_3)) | ([3](#_ENREF_3)) |
| Lck | Lck-GFP ([5](#_ENREF_5)) | ([12](#_ENREF_12)), S14 video |
| LFA-1 | ICAM-1-GFP ([48](#_ENREF_48)) | ([3](#_ENREF_3)) |
| Ly108 | Ly108-1-GFP ([3](#_ENREF_3)) | ([3](#_ENREF_3)) |
| Moesin | Moesin-GFP * | S2F Fig., S15 video |
| Myosin light chain kinase | MLCK-YFP-CaM-CFP (K. Kamm) | S2G Fig., S16 video |
| Myosin 1C | Myosin 1C-GFP ([5](#_ENREF_5)) | Fig. 4C, S17 video |
| Myosin II RLC | Myosin II RLC-GFP * | Manuscript in preparation |
| Nck | Nck-GFP (C. Freund) | ([12](#_ENREF_12)), S18 video |
| NFκB p65 | GFP-p65 (L. Schmitz) | Fig. 4D, S19 video |
| PI 3-kinase | PI3K p85 SH2-interSH2-SH2-GFP ([3](#_ENREF_3)) | ([12](#_ENREF_12)), ([3](#_ENREF_3)) |
| PIP_2_ | GFP-PLCδ-PH ([3](#_ENREF_3)) | Fig. 1F, video in ([3](#_ENREF_3)) |
| PIP_3_ | GFP-Cytohesin PH ([3](#_ENREF_3)) | ([3](#_ENREF_3)) |
| PIP 5-kinase α | PIP 5-kinase α ([49](#_ENREF_49)) | ([49](#_ENREF_49)) |
| PIP 5-kinase γ87 | PIP 5-kinase γ87-GFP ([49](#_ENREF_49)) | ([49](#_ENREF_49)) |
| PIP 5-kinase γ90 | PIP 5-kinase γ90-GFP ([49](#_ENREF_49)) | ([49](#_ENREF_49)) |
| α-Pix | GFP-α-Pix ([5](#_ENREF_5)) | S2H Fig., S20 video |
| PKCη | PKCη -GFP ([3](#_ENREF_3)) | ([3](#_ENREF_3)) |
| PKCθ | PKCθ -GFP ([3](#_ENREF_3)) | ([3](#_ENREF_3)) |
| PKCζ | PKCζ-GFP * | S2JI Fig., S21 video |
| PLCγ | PLCγ PH-SH2-SH2-GFP ([3](#_ENREF_3)) | ([12](#_ENREF_12)) |
| Rac | POSH GBD-GFP-CAAX ([3](#_ENREF_3)) | ([3](#_ENREF_3)) |
| Rho | Rhotekin GBD-GFP-CAAX ([3](#_ENREF_3)) | ([3](#_ENREF_3)) |
| Rltpr | Rltpr-YPet ([50](#_ENREF_50)) | ([50](#_ENREF_50)) |
| R-pre | GFP-R-pre ([51](#_ENREF_51)) (S. Grinstein) | S2J Fig., S22 video |
| SHP-1 | SHP-1-GFP * | Fig. 8D, S23 video |
| SKAP55 | GFP-SKAP55 (C. Freund) | Fig. 4B, manuscript in preparation |
| SLAT | GFP-SLAT ([5](#_ENREF_5)) | S2K Fig., S24 video |
| SLP-76 | SLP-76-GFP ([5](#_ENREF_5)) | Fig. 1E, S1 video |
| TCR as MHCII on the APC | I-E^k^-GFP ([3](#_ENREF_3)) | ([3](#_ENREF_3)) |
| TCR as CD3ε | CD3ε-GFP ([3](#_ENREF_3)) | ([3](#_ENREF_3)) |
| TCR as TCRζ | TCRζ-GFP ([3](#_ENREF_3)) | ([3](#_ENREF_3)) |
| Tec | Tec PH-TH-SH3-SH2-GFP * | ([3](#_ENREF_3)) |
| Themis | Themis-GFP ([22](#_ENREF_22)) | ([22](#_ENREF_22)) |
| VASP | GFP-VASP * | S2L Fig., S25 video |
| Vav1 | Vav1-GFP ([5](#_ENREF_5)) | S2 video, ([12](#_ENREF_12)) |
| WASH | GFP-WASH * | S2M Fig., S26 video |
| WASP | GFP-WASP * | Manuscript in preparation |
| WAVE-2 | GFP-WAVE2 ([3](#_ENREF_3)) | Manuscript in preparation |
| WDR34 | WDR34-GFP * | S2N Fig., S27 video |
| ZAP-70 | ZAP-70 tandem SH2-GFP ([3](#_ENREF_3)) | ([3](#_ENREF_3)) |
